# DNA damage in human sperm: The sperm chromosome assay

**DOI:** 10.1002/rmb2.12461

**Published:** 2022-04-20

**Authors:** Seiji Watanabe

**Affiliations:** ^1^ Department of Anatomical Science Hirosaki University Graduate School of Medicine Hirosaki Japan

**Keywords:** aging sperm, DNA damage, DNA repair, structural chromosome aberrations

## Abstract

**Background:**

Sperm DNA damage is a major cause of pre‐ and post‐implantation embryonic loss in humans. However, the factors that control how and when such DNA damage occurs in human sperm are poorly understood.

**Methods:**

Here, I review information relating to sperm DNA damage that can be obtained from the sperm chromosome assays described in the existing literature.

**Main findings:**

The sperm chromosome assays, which consist of interspecific in vitro fertilization or intracytoplasmic sperm injection using murine oocytes and subsequent chromosome analysis, indicate that the proportion of sperm showing DNA damage is initially low and there are larger numbers of sperm with potential membrane and DNA damage that are induced after ejaculation and separation from the seminal plasma. Other assays that directly detect sperm DNA (e.g., TUNEL assays, Comet assays, and acridine orange test) are not able to distinguish and detect the initial and potential DNA damage. Furthermore, the positive values in these direct assays are influenced by the frequency of immotile sperm and amorphous sperm populations.

**Conclusion:**

The findings in the sperm chromosome assays show that further improvements in sperm preparation protocols may result in the reduction of sperm DNA damage, followed by more successful outcomes in infertility treatment.

## Graphical abstract

This review explains with the data reported by studies with the sperm chromosome assays how and when DNA damage, which contributes to pre‐ and post‐implantation embryonic loss, occurs in human sperm. The sperm chromosome assay is superior to other assays that directly detect sperm DNA damage (TUNEL assays, Comet assays, and the acridine orange test).

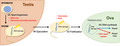

## 1 INTRODUCTION

**FIGURE 1:**
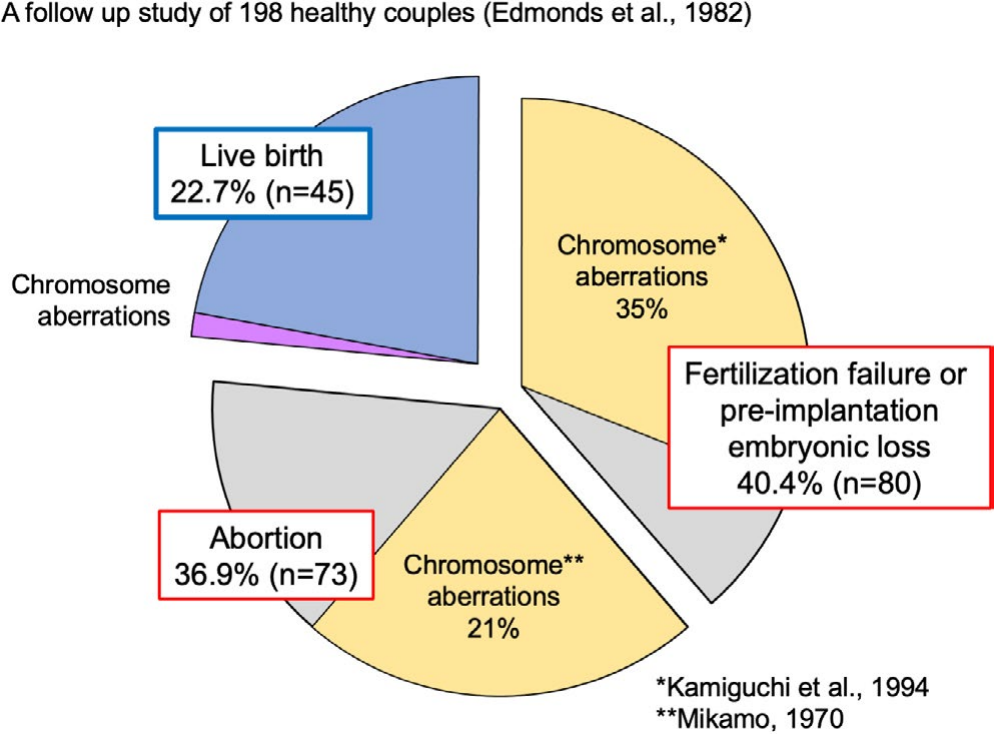
Reproductive efficiency in humans. The frequencies of fertilization failure or pre‐implantation embryonic loss and post‐implantation embryonic loss (abortion), as calculated from data acquired by a follow‐up study of 198 healthy couples^1^

**FIGURE 2:**
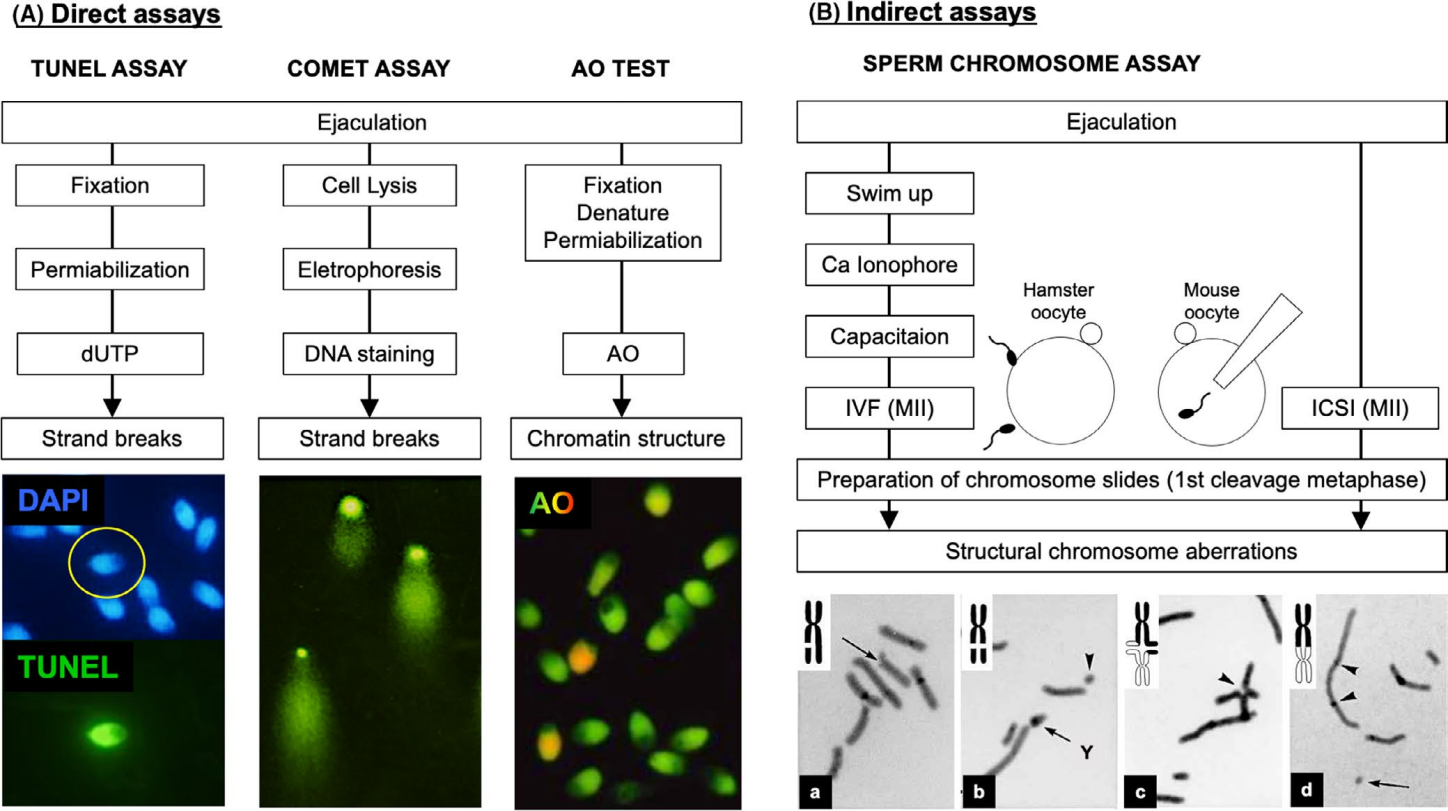
Methods to evaluate human sperm DNA and chromatin damage. A summary of the procedures used for direct assays (TUNEL assay, Comet assay, and acridine orange test (AO) test) and indirect assays (the sperm chromosome assay). In direct assays, sperm are directly stained with fluorescent regents that react with DNA molecules and then evaluated under a fluorescence microscope (A). The sperm chromosome assay is classified as an indirect assay and requires IVF or ICSI between human sperm and murine oocytes to analyze chromosomes at the first mitotic metaphase. Experience is required for the correct analysis of the sperm chromosome complements when stained with Giemsa and examined by microscopy (B). A photograph of the Comet assay is reproduced with permission by Dr Kusakabe (Department of Biology, Asahikawa Medical School). Photographs of chromosomes are reproduced from my previous study^11^

It is well known that many embryos and fetuses are lost during pregnancy in humans. Edmonds et al.^1^ analyzed 198 healthy women with hormonally confirmed ovulation and coitus; only 22.7% of these women successfully produced a baby, since many embryos or fetuses failed to implant on the uterine wall (40.4%) or failed to be delivered (36.9%), respectively (Figure 1). Many cytogenetic studies have shown that the main cause of early embryo loss in humans is chromosomal abnormalities in both the male and female gametes^2^, ^3^; these can be classified into numerical and structural chromosomal aberrations (Figure 2B, a–d). Although the number of babies arising from assisted reproductive technologies (ART) is increasing globally, it is very difficult to overcome problems relating to embryonic chromosomal abnormalities.

This review focuses on the characteristics of DNA damage in human sperm; this damage can subsequently be transformed into structural chromosome aberrations in early embryos. I also focus on cytogenetic assays that can be used to detect such DNA damage. This is important because it has been estimated that the contribution of sperm DNA damage to the loss of human embryos was greater than that of sperm aneuploidy (14.1% vs. 1.4%^3^, ^4^). In addition, DNA damage is more frequent in sperm when compared with oocytes (4.7%^3^), thus indicating that the mechanisms underlying such damage are complex and dependent on sperm‐specific characteristics, although numerical chromosomal aberrations are produced with similar mechanisms (pre‐division and non‐disjunction) during meiosis in both sperm and oocytes.^5^

## 2 METHODS TO STUDY SPERM DNA DAMAGE

Two types of cytogenetic methods are available for the evaluation of genetic integrity in mature sperm, as shown in Figure 2. One type is a direct assay in which ejaculated sperm or sperm collected from the testis or epididymis are used directly to evaluate the integrity of their chromatin structure or DNA (Figure 2A). These tests are easy to be performed in clinical laboratories and can estimate the cytogenetic quality of human semen samples. However, the positive detection of these tests, which relate to a portion of a whole sperm population in each semen sample, can depend on sperm motility rates and morphologically abnormal sperm rates.^6^, ^7^, ^8^, ^9^, ^10^ It has been proven that the DNA of immotile dead sperm and sperm with severely morphological aberrations in the head is more frequently damaged than that of motile sperm with a normal‐shaped head.^11^, ^12^, ^13^ In addition, some sperm tests actively denature DNA and protein to enhance the reactivity of the test reagents; accordingly, test results may overestimate the genetic risk of the sperm samples being assayed. Many previous reports have evaluated sperm assays in significant detail.^14^, ^15^, ^16^, ^17^, ^18^, ^19^, ^20^ Consequently, in this review, I will only provide brief explanations relating to the important points of these tests in order to facilitate a better understanding of the results obtained by the most commonly used direct assays (e.g., TdT‐mediated dUTP Nick End Labeling (TUNEL) assay, Comet assay, and acridine orange test).

Another type is an indirect assay, such as the “sperm chromosome assay” (Figure 2B). In the sperm chromosome assay, all human sperm chromosomes were constructed and fully analyzed in murine oocytes when they reached to the first cleavage metaphase after the penetration of human sperm by in vitro fertilization (IVF) or intracytoplasmic injection (ICSI).^3^, ^13^, ^21^, ^22^ Although these assays require a high degree of skill to be performed, they also enable the detection of DNA breaks more precisely and allow us to investigate their relationship with cytological characteristics in the sperm. Since previous reviews on sperm DNA damage have not dealt with findings obtained from the sperm chromosome assay, this manuscript focuses predominantly on findings that have been reported by studies using the sperm chromosome assay in comparison with data derived from direct assays. In this way, I hope that this review will provide clinicians and researchers with a deeper understanding of DNA damage in sperm.

### 2.1 Direct analysis of sperm DNA damage

#### 2.1.1 The TUNEL assay

**FIGURE 3:**
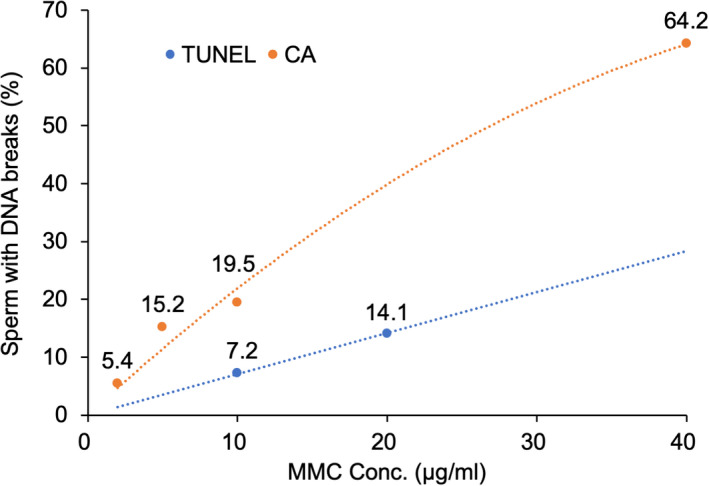
The distinct sensitivities of two sperm DNA assays. DNA breaks induced by Mitomycin C (MMC) in normal‐shaped sperm were detected by the TUNEL assay (TUNEL) and sperm chromosome assay (CA) and the incidences of sperm with DNA breaks were compared between the two methods. Fluorescent TUNEL‐positive sperm were identified and counted on graphic images captured by a laser scanning microscope.^24^ In the sperm chromosome assay, sister chromatid breaks in the chromosome complement of human sperm which had penetrated into hamster oocytes were counted under a light microscope after Giemsa staining (my own unpublished data)

**FIGURE 4:**
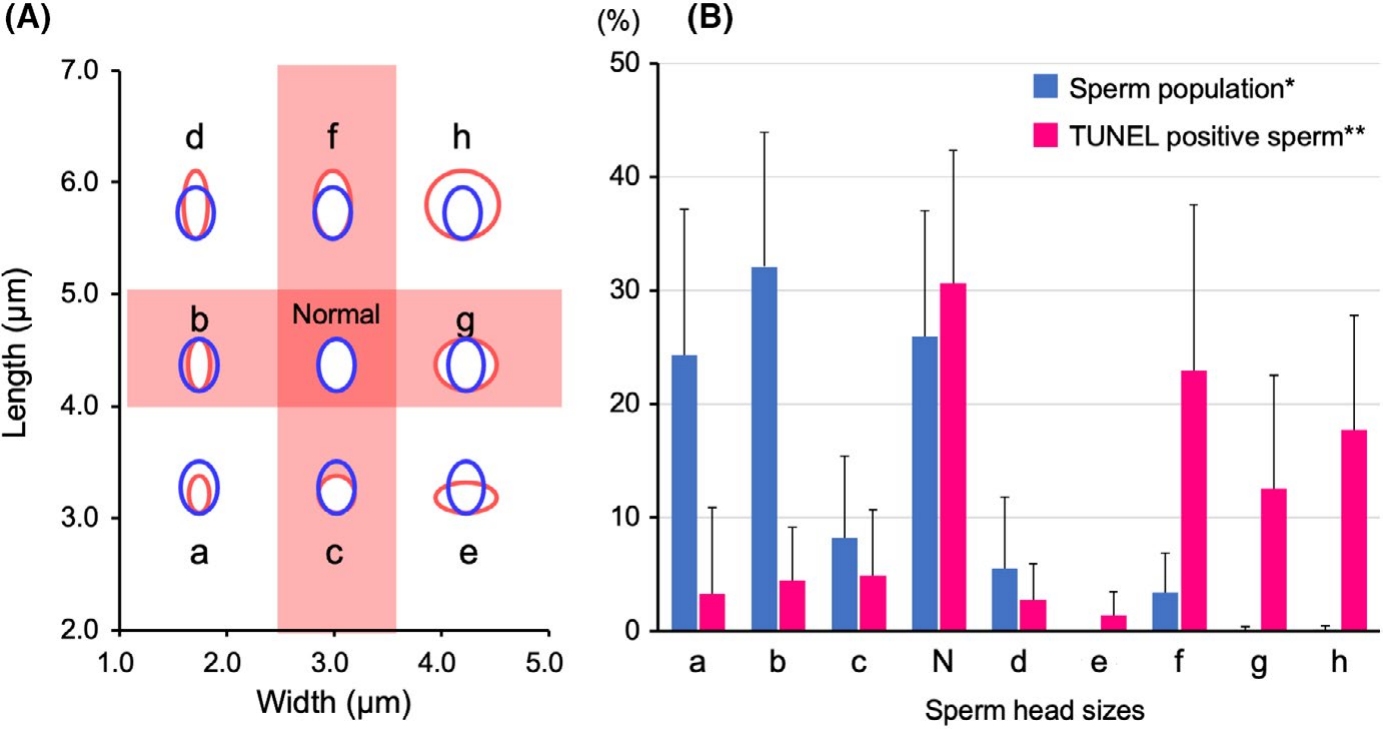
Relationship between sperm head sizes and DNA damage. Sperm were classified into nine groups (a–h and normal) based on their head width and length (A). Blue circles show the head shape of morphologically normal sperm (WHO criteria) while red circles represent the head shape of each morphologically abnormal sperm (a–h). To calculate the population of each head size group (B), the head width and length of 100 randomly selected sperm were measured and classified into nine groups (*). The head sizes of 100 TUNEL‐positive sperm were also measured and classified into nine groups (**). Populations of sperm that were classified into normal, a and b groups were predominant in 21 normozoospermic semen samples, although the risk of DNA damage was approximately five times higher in the normal group than in the a or b groups^32^

The TUNEL assay enables us to detect DNA strand breaks in sperm nuclei under microscopy by labeling the DNA with fluorescein dUTP which is enzymatically added with terminal deoxyribonucleotidyl transferase (TdT).^23^ My previous study, using fluorescent microscopy, showed that this assay could detect a dose‐dependent increase of DNA breaks in human sperm exposed to Mitomycin C (MMC),^24^ thus indicating its reliability as an evaluation method for DNA damage. However, the sensitivity of the TUNEL assay, which is measured with the frequency of cells with DNA damage, appears to be two‐fold lower when compared with the sensitivity of the sperm chromosome assay. The sperm chromosome assay was shown to detect DNA damage in 19.5% of sperm that were exposed to 10 µg/ml of MMC for 2 h and successfully fertilized hamster oocytes. Using the TUNEL assay, 7.2% of sperm of the whole sperm population were positive after exposure to the same dose of MMC (Figure 3, my own unpublished data). Therefore, there is a probability that some of the sperm DNA breaks are not efficiently labeled with fluorescein dUTP by TdT. In my laboratory, ejaculated sperm were fixed with 4% paraformaldehyde to minimize an increase of DNA damage during sample preparation. In previous studies involving the TUNEL assay, the binding of dUTP to the sites of DNA breaks was accelerated by denaturing the sperm nuclear protamine and DNA molecules with acetic acid. The denaturing process increases the sensitivity of this assay (i.e., the frequency of sperm that are positive for DNA breaks).^25^, ^26^, ^27^, ^28^, ^29^, ^30^, ^31^ An important point to consider when applying the TUNEL assay is to understand that the denaturing process shows bias toward sperm with a lower nuclear maturity; previous data were significantly correlated to the proportion of sperm with a morphologically abnormal head. On the other hand, this assay does not require the digestion of nuclear proteins; therefore, the shape of the sperm head is maintained until final observation. This process allows for the efficient examination of the relationship between sperm head morphology and DNA breaks (Figure 4).^32^, ^33^ When using flow cytometry for the TUNEL assay, it appears to be necessary to establish a strictly standardized protocol.^34^

#### 2.1.2 The Comet assay

The Comet assay visualizes fragmented DNA by electrophoresis as comet tail‐like fluorescence that can be detected by simple fluorescence microscopy (Figure 2). In this assay, sperm nuclei are freed from the plasma membrane and nuclear proteins by a lysis solution; dithiothreitol is used to break the protamine disulfide bonds and allow sperm head swelling. During this lysis process, the original shape of the sperm head undergoes alterations and it becomes impossible to analyze the relationship between DNA damage and sperm head morphology. In this assay, DNA fragments are moved away from the denuded swollen sperm DNA mass by electrophoresis under neutral or alkaline condition, thus forming a tail that is similar to a comet.^16^, ^35^, ^36^ It is understood that alkaline Comet assay detects both single‐ and double‐stranded DNA breaks and neutral Comet assay detects mostly DNA double‐strand breaks.^16^, ^37^, ^38^ This assay generally evaluates DNA damage in a single sperm by measuring the specific length of the comet tail, which significantly depends on the electrophoresis protocols used. In other words, when we prove whether or not a single sperm is genetically damaged, the length of the tail must be compared to negative and positive control sperm (exposed to mutagenic chemicals or radiation). Therefore, this assay provides us with a useful option to compare the frequencies of sperm with DNA damage between two semen sample groups, although it is hard to quantify DNA breaks precisely in each sperm. The detection sensitivity (the frequency of sperm with DNA damage) is approximately two‐fold lower in the alkaline Comet assay than the sperm chromosome assay; these assays were previously used to detect DNA damage in approximately 50% of human sperm exposed to 200 or 100 µg/ml of MMS, respectively.^39^, ^40^ Many of the previous studies that used the comet assay only identified a relationship between sperm DNA damage with sperm motility and abnormal head morphology. Since these semen parameters can be measured under a microscope in routine semen analysis, it seems difficult to find any reason to add the comet assay to one of the routine semen tests in ART treatment. Ribas‐Maynou et al.^41^ reported that neutral and alkaline Comet assays offer the possibility of differentiating single‐ and double‐stranded DNA breaks, respectively. However, there is a question on the possibility, since it is considered that alkaline Comet assays, which detect both single‐ and double‐stranded DNA breaks, are more sensitive to DNA damage.

#### 2.1.3 The acridine orange test (AO test)

The AO test estimates sperm DNA damage in a different manner from the two direct assays described above. The AO test was first applied to human sperm by Tejada et al.^42^ In this test, mature sperm are fixed with acetic alcohol; this increases membrane permeability and denatures nuclear proteins; this is followed by AO treatment (Figure 2). Since sperm DNA is tightly condensed with an abundance of disulfide bonds between sperm‐specific nuclear proteins (protamine), AO monomers are inserted into the grooves between the two base pairs of DNA molecules (intercalation); subsequently, these monomers can emit green fluorescence under a fluorescent microscope. However, at the sites where sperm DNA is denatured by acid treatment, AO dimers are allowed to enter the DNA groves and emit red fluorescence.^43^, ^44^ In another words, AO can make it possible to distinguish between the groves of DNA strands that remain tightly closed and those that are wide open after the denaturation process. Therefore, red fluorescence does not necessarily appear to prove the existence of DNA strand breaks. The mechanisms that occur between AO molecules and DNA strands indicate that the findings of Chohan et al.^45^ are reasonable; these authors reported that different fixation procedures can affect the proportion of green‐fluorescent sperm. When AO staining was conducted after fixation with paraformaldehyde, the sperm exhibited yellow, orange, or red fluorescence, which were considered as damaged cells, significantly decreased compared to those fixed with acetic alcohol (Carnoy's solution). A chromatin structure of sperm that have undergone excessive fixation becomes resistant to denaturing agents, thus resulting in the reduced accessibility of AO molecules to DNA. Consequently, the AO test is considered to visualize sperm with fragile DNA sites that may be prospectively transformed to DNA breaks due to oxidative or osmotic stresses during sperm movement. Accordingly, there is a possibility that the AO test may overestimate DNA damage in human sperm. In addition, it must be considered that AO itself is a very toxic molecule that can induce DNA breaks.^43^ In Chinese hamster somatic cells exposed to acridine, the incidence of cells with structural chromosome aberrations was found to be 22%.^46^ I have previously attempted chromosome analysis in fresh human sperm with green and red fluorescence to confirm whether distinct colors are related to DNA breaks. I found that the human sperm chromosome assay detected multiple structural chromosome aberrations in sperm exposed to AO (my own unpublished data). On the other hand, Hoshi et al.^47^ reported that more sperm with green fluorescence were frequently bound to the surface of the zona pellucida than sperm with yellow or red fluorescence, thus resulting in higher fertilization rates in IVF. This result indicates a possibility that even if there are statistically significant differences in ART outcomes between two semen sample groups showing distinct AO test data, sperm DNA damage may not be responsible for the final consequence. Therefore, the AO test should be applied to monitor temporal changes in the frequency of good quality sperm, which are stained green, while considering the characteristics described above.

### 2.2 Indirect analysis of sperm DNA damage

#### 2.2.1 The human sperm chromosome assay

When a sperm penetrates an oocyte, the sperm nuclear plasma membrane disappears and sperm DNA, which had previously been tightly folded by the sperm‐specific nuclear protein, protamine,^48^, ^49^ is spread and duplicated in the male pronucleus. Subsequently, the DNA condenses to form chromosomes at the first mitotic metaphase. If there is damage in the DNA of the fertilizing sperm, then this can be viewed as a structural chromosomal aberration(s) under a microscope (Figure 2B).

It is not ethical to collect and use 1‐cell human embryos for experimental research. Therefore, researchers have attempted to use unfertilized murine oocytes as an alternative to human oocytes and inseminate them with human sperm in vitro. In a previous study, Yanagimachi^50^ found that zona‐free Syrian hamster oocytes could be penetrated by the sperm of several mammalian species. Kamiguchi and Mikamo^21^ adopted these oocytes to investigate chromosomes in human ejaculated sperm. Prior to their study, the Tarkowski method was generally used for the preparation of chromosome slides from mammalian oocytes.^51^ However, this method cannot avoid the artificial loss of chromosomes arising during the process in which the plasma membrane is punctured by dropping a fixative medium on the oocytes. In the gradual fixation‐air drying method developed by Kamiguchi and Mikamo,^21^ oocytes are gradually fixed and flattened on glass slides, thus avoiding puncture of the plasma membrane. Accordingly, a human sperm chromosome assay was established that could evaluate sperm DNA integrity in an efficient manner.

In the human sperm chromosome assay, sperm are exposed to 5–15 µM of Ca^2+^ ionosphere to induce the acrosome reaction and then co‐incubated with zona‐free hamster oocytes. This assay only targets motile sperm that have the potency to undergo the acrosome reaction and activate oocytes. However, there is a regrettable point in this assay in that it cannot determine the morphological features of the sperm that penetrate hamster oocytes. Therefore, it has not been clear whether human sperm with amorphous heads are able to penetrate oocytes in a spontaneous manner.

Following IVF, intracytoplasmic sperm injection (ICSI) was developed to treat cases of severe male factor infertility who repeatedly fail to generate fertilized embryos due to very poor semen quality. Kimura and Yanagimachi^52^ introduced piezo‐ICSI for the penetration of mouse oocytes, which are more resistant to micromanipulation than Syrian hamster oocytes. In previous studies, piezo‐ICSI was combined with the human sperm chromosome assay to allow us to analyze relationships between chromosome aberrations and several sperm morphological and physiological characteristics (morphology, motility, and plasma membrane integrity).^11^, ^12^, ^13^, ^22^, ^24^ The sensitivity of detection for the sperm chromosome assay is higher than the sensitivity of the direct assays described earlier.

## 3 CHARACTERISTICS OF STRUCTURAL CHROMOSOME ABERRATIONS IN HUMAN SPERM

According to previous studies involving the sperm chromosome assay, the spontaneous incidence of chromosome aberrations in human sperm from normozoospermic donors was 14.1%^3^; this value was much higher than that of murine sperm fertilized in vivo. In addition, a large individual difference was detected with regards to sperm DNA damage. These results showed that DNA damage occurs frequently before fertilization in human sperm and is dependent on a range of unknown and individual factors. Kamiguchi et al.^3^ found no relationship between smoking or drinking alcohol and the rates of structural chromosome aberrations. The chromosome aberrations detected by the sperm chromosome assay can be classified into the following four types: (a) chromatid breaks, (b) chromosomal breaks, (c) chromatid exchanges, and (d) chromosomal exchanges (Figure 2B). The dominant aberration type is chromosomal breaks (60.3%^22^; 75.8%^53^; 71.1%^54^); these breaks are derived from a double‐stranded DNA break. This fact provides us with important information relating to how DNA damage in the human sperm nucleus is converted into structural chromosome aberrations in the cytoplasm of the oocyte. Since a developing sperm cell discards DNA repair enzymes along with almost all of its cytoplasm during spermiogenesis,^55^ DNA damage tends not to be repaired and accumulates until fertilization. The production of chromosomal breaks implies that complementary sites to the DNA sites containing a single‐stranded break are not correctly repaired and duplicated during the S phase in oocytes. Although the precise mechanism responsible for this phenomenon have yet to be elucidated, most oocytes that are fertilized with sperm containing DNA damage appear to select cell death by leaving the sperm DNA damage unrepaired.

## 4 SPERM DNA DAMAGE AND TRANSFORMATION

**FIGURE 5:**
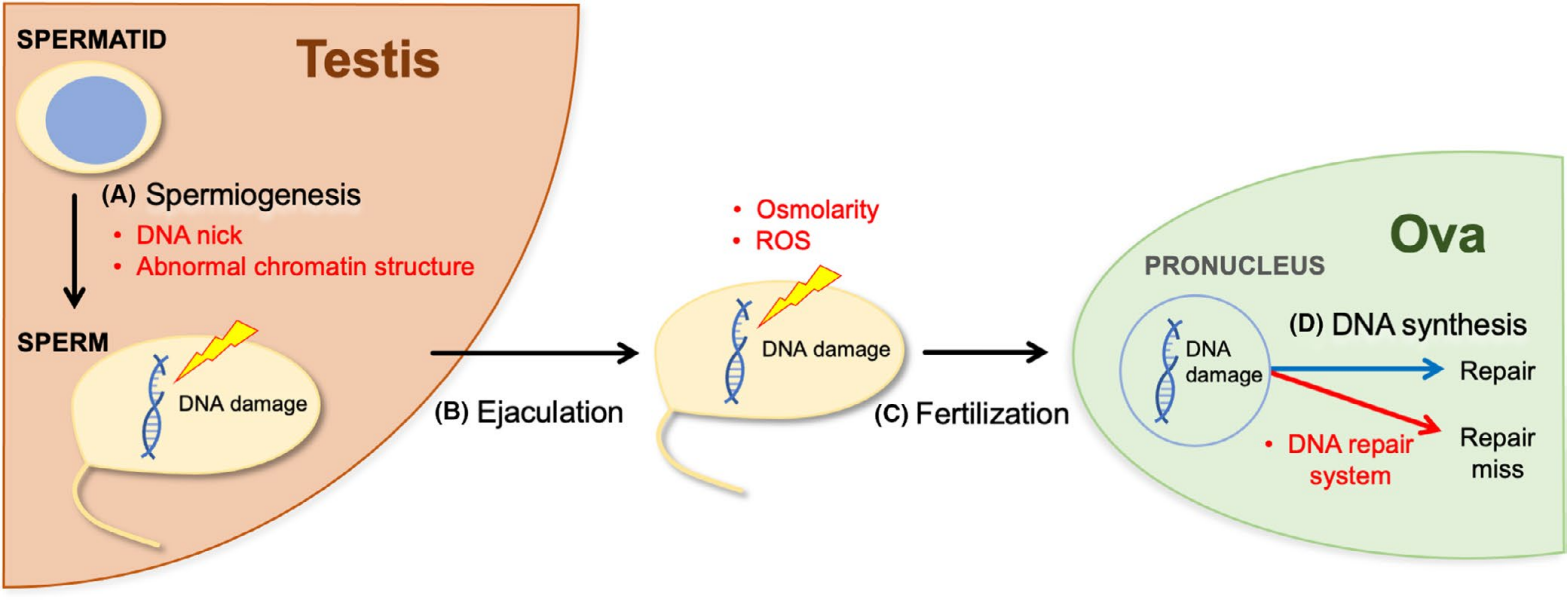
The production of structural chromosome aberrations in sperm. Factors that affect sperm DNA (annotated by red letters) are shown from spermiogenesis to post‐fertilization DNA synthesis (A–D). The damage accumulated in sperm DNA before fertilization is finally transformed to structural chromosome aberrations through the DNA repair system in the oocyte cytoplasm

Figure 5 shows possible opportunities for when sperm DNA damage occurs and a pathway by which sperm DNA damage transforms to structural chromosome aberrations. Following the completion of meiosis, the spermatids mature in the testis throughout spermiogenesis (Figure 5A). The resultant mature sperm are subsequently ejaculated from the male genital tract into the female vagina (Figure 5B). One of the ejaculated sperm reaches and penetrates an oocyte within the ampulla region of the oviduct in vivo (Figure 5C) and forms a male pronucleus, in which the sperm DNA undergoes duplication (Figure 5D). For the treatment of male infertility, sperm are collected by ejaculation and used to penetrate oocytes in vitro. During all of these steps, there is a risk of DNA damage in the sperm nuclei (in the elongated spermatids, mature sperm, and male pronucleus). Sperm DNA damage caused during these steps is then converted to structural chromosome aberrations at the first mitotic cleavage.

### 4.1 Spermiogenesis

Following meiosis, spermatids are freed from most of the cytoplasm and their nuclei condense owing to the gradual replacement of somatic nuclear proteins (histone) with sperm‐specific lysine‐rich nuclear proteins (protamine), which are bound with each other by disulfide bonds; this allows transformation into the mature sperm^49^, ^56^ (Figure 5A).

#### 4.1.1 DNA nicks

**FIGURE 6:**
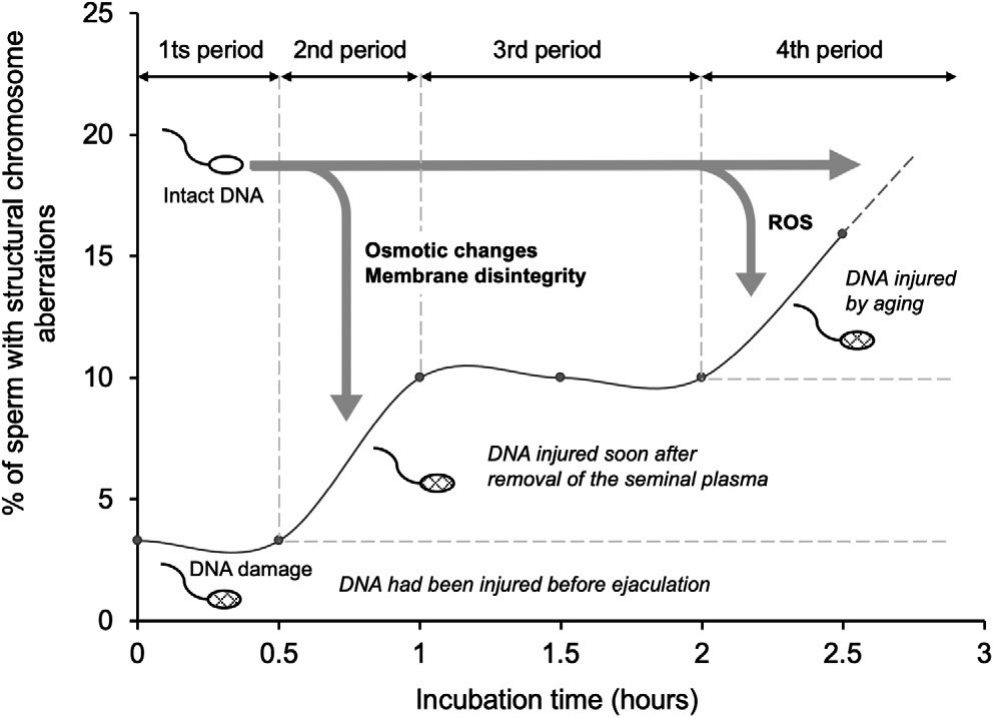
Increased structural chromosome aberrations in human sperm in vitro. Time‐dependent increase of structural chromosome aberrations in sperm with normal‐shaped heads (3 µm × 4–5 µm) and motility, as illustrated by a line graph.^12^, ^22^, ^33^ DNA damage in the normal sperm was rare (3%) 30 min after removal of the seminal plasma (the 1st period). Within the next 30 min, DNA damage occurred rapidly in response to the influx of hypo‐osmotic solution in some sperm in which the plasma membrane had been potentially damaged (the 2nd period). For the next 1 h, there was no increase in the incidence of sperm with DNA damage, thus suggesting that the DNA of sperm with good levels of integrity in the plasma membrane was stable during this period (the 3rd period). When the 2 h passed after removal of the seminal plasma, sperm DNA began to incur damage again as a result of the effect of ROS production (the 4th period); this was consistent with alterations of sperm movement (hyperactivation)

DNA nick formation during spermiogenesis is one of the possible origins of the structural chromosome aberrations that are responsible for pre‐ and post‐implantation embryo loss in humans. In a previous study, Smith and Haaf^57^ reported that DNA nicks were found in murine elongated spermatids but not in spermatocytes and spermatids when detected by fluorescence in situ end labeling. Marcon and Boissonneault^58^ and Laberge and Boissonneault^59^ also observed that the incidence of DNA nicks significantly decreased with sperm maturation in the murine model. These studies suggest that the temporal appearance and disappearance of DNA nicks is a collateral phenomenon of the significant structural rearrangements of chromatin during spermiogenesis. In addition, spermatids also discard their DNA repair enzymes, which are proteins that can respond and recover the abnormal position of the DNA damage,^60^, ^61^, ^62^, ^63^, ^64^ along with their cytoplasm during this period. Therefore, the DNA nicks and other forms of DNA damage that are subsequently produced in the testis, seminal ducts, and female genital tracts, can accumulate until fertilization; at this point, the cytoplasm of the oocyte provides ooplasmic DNA repair enzymes (Figure 5D). However, in the mouse, the DNA nicks in spermiogenesis are not responsible for early embryonic loss; this is because the frequency of spontaneous structural chromosomal aberrations is extremely low at the 1‐cell stage, both in vivo and in vitro (1.76% in vivo and 1.74% in vitro^65^; 0.4%^66^; 2%^67^, ^68^), thus suggesting that almost all DNA nicks undergo repair in mouse oocytes. Furthermore, in the Chinese hamster, sperm DNA is rarely damaged in vivo (1.4%^69^). In contrast, the incidence of sperm with structural chromosome aberrations in the Syrian hamster was comparably higher in vivo (8.3%^70^; 6.9%, my own unpublished data), when compared with that in other murine species and the human. Therefore, it is suspected that more DNA nicks are spontaneously transformed to structural chromosome aberrations in the Syrian hamster. As with mouse and Chinese hamster sperm, the contribution of DNA nicks to structural chromosome aberrations appears to be limited in human sperm; I estimated this to be approximately 3% in normozoospermic semen samples (Figure 6),^33^ as described in Section 4.2.

#### 4.1.2 Abnormal sperm head condensation

Human ejaculated semen contains many sperm with morphologically aberrant heads; these are the result of abnormal chromatin condensation during spermiogenesis. An elongated head is one of the abnormal sperm head morphologies and is determined by a longer head length (length > 5 µm and normal width) according to the WHO criteria.^71^ My previous cytogenetic study^11^ showed that DNA was extremely fragmented in 33.3% of sperm with elongated heads. It is interesting that only elongated sperm heads are commonly associated with DNA damage; in contrast, sperm with large heads (length >5 µm and width > 3.5 µm) showed no significant increase in structural chromosome aberrations (8.3%). Therefore, this abnormal pattern of chromatin condensation, involving an elongated head shape (length/width proportion > 2), appears to be involved in the mechanism by which more DNA nicks form during spermiogenesis and subsequently maintained and transformed to structural chromosome aberrations after fertilization. Double‐stranded breaks are transiently formed according to histone hyperacetylation in the elongating mouse spermatid undergoing an increase in the nuclear length/width proportion.^59^ Elongated human sperm may have an immature chromatin structure that is similar to that of elongating mouse spermatids. On the other hand, I noticed that the incidence of structural chromosome aberrations was slightly lower in sperm with smaller heads (approximately 3 × 3 µm) than in sperm with normal heads.^11^ Martin et al.^72^ also reported that sperm with small heads in infertile males with globozoospermia were cytogenetically normal, although they lacked an acrosome. Considering these results, I hypothesized that smaller heads are the result of excessive chromatin condensation with rich disulfide bonds and that such tightly condensing chromatin structures are more resistant to the formation of DNA breaks. Figure 4 shows the relationship between sperm head sizes and DNA damage, as detected by the TUNEL assay.^32^ As I expected, the risk of DNA damage was approximately five‐fold lower in sperm with smaller heads (small heads (a) or narrow heads (b)) than sperm with normal heads (N), thus suggesting the probability that sperm with normal heads are not necessarily the most suitable to be selected for ICSI treatment in terms of genetic integrity.

#### 4.1.3 Environmental mutagens

The other forms of sperm DNA damage that can be formed during this period are single‐ and double‐stranded DNA breaks that are induced by environmental mutagens such as radiation and chemical substances. Radiation directly breaks DNA strands in a linear dose‐dependent manner in both human and murine sperm.^53^, ^68^, ^70^ Chemical mutagens that are administered as chemotherapies, or consumed along with food, can also cause strand breaks in sperm DNA directly or indirectly after metabolism in the liver tissues.^38^, ^54^, ^73^, ^74^ These results indicate that the sites of DNA breaks which are newly formed in mature sperm exposed to radiation and chemicals are not commonly repaired by oocyte DNA repair enzymes. No clastogenic effect has been observed in human sperm exposed to microwave radiation,^75^ low‐frequency electromagnetic fields,^76^ and dioxins^77^ in vitro.

#### 4.1.4 Temperature of the environment surrounding the testis

Some studies have reported that people who regularly cycle, undergo physical training, or work with bicycles for the public traffic transport, have a higher concentration of morphologically aberrant sperm in low‐quality semen samples.^78^ These problems appear to result from testicular heat stress.^79^ Of the several types of morphologically abnormal sperm heads, sperm with elongated heads appear to be able to penetrate oocytes in vivo; this is because the sperm obtained from normozoospermic semen samples possess preserved acrosomes.^11^, ^24^, ^32^ Sperm with severe teratogenic heads, which are known to be significantly related to structural chromosome aberrations,^13^ cannot penetrate oocytes efficiently. Therefore, an increase in such abnormalities could easily affect the success of fertilization rates in vivo.

### 4.2 Ejaculation

In mammalian species, sperm are ejaculated into the female vagina (Figure 5B). Consequently, it is very difficult to investigate DNA damage in the sperm that are moving to the oviducts. Therefore, studies of mammalian sperm have been generally carried out by in vitro culture. In the Syrian hamster, the insemination of sperm in vitro caused a significantly higher incidence of structural chromosome aberrations at the first cleavage metaphase, as compared with sperm inseminated in vivo (20.5% vs. 6.9%, respectively, my own unpublished data). This result indicates that sperm DNA is significantly affected by the environment in which the sperm is ready for oocyte penetration.

#### 4.2.1 Seminal plasma

Mature human sperm that have been stored in the testis or genital tracts are mixed with the seminal plasma upon ejaculation.^80^ In vitro, ejaculated human semen with high levels of viscosity and higher osmolarity^12^, ^81^ is gradually liquified over a 30‐min period at 37°C. My previous studies revealed that the seminal plasma protected the sperm plasma membrane, effectively preventing potential chromatin structure damage from being converted into actual DNA strand breaks.^12^, ^33^ When using the sperm chromosome assay with ICSI, the incidence of structural chromosome aberrations in sperm that were freed from the seminal plasma was three times higher than that in sperm stored in the seminal plasma for a few hours after ejaculation (Figure 6, 8% vs. 3%, *p *< 0.05). Therefore, in healthy men, it is considered that mature sperm with actual DNA strand breaks are spontaneously very low. Furthermore, even in immotile dead sperm with membrane damage, the integrity of DNA was retained (97%) for at least 3 h in the seminal plasma.^12^, ^33^ On the other hand, superoxide dismutase (SOD), which metabolizes reactive oxygen species (ROS) and is contained in the seminal plasma, also appears to play a role in preventing an increase in sperm DNA breaks. Because concentrations of SOD correlate with human semen qualities.^82^, ^83^, ^84^ In contrast to the benefits of the seminal plasma, a previous paper reported that seminal hyper‐viscosity was associated with a success rate with regard to in vitro fertilization and embryo transfer.^85^ The authors of this paper postulated that DNA damage or abnormal chromatin structures played a role in these distinct outcomes. When viscous semen samples were used for the sperm chromosome assay, I noticed a tendency that sperm required higher concentrations of Ca^2+^ ionophore (15 µM) to induce hyper‐activation^86^ and the acrosome reaction for IVF with zona‐free hamster oocytes; interestingly, their motility declined earlier (my own unpublished data). Therefore, it is suspected that the DNA of sperm from viscous semen receives more damage from intracytoplasmic physiological changes that occur during capacitation.

#### 4.2.2 Osmolarity

After removal of the seminal plasma, there seem to be several periods in which alteration of the sperm chromatin structure can be caused by environmental and physiological factors (Figure 6). In my studies with the human sperm chromosome assay, the incidence of structural chromosome aberrations was 3% in sperm that were directly selected and examined soon after ejaculation (within 30 min: Figure 6, the 1st period). Thirty minutes after removal of the seminal plasma, the incidence of structural chromosome aberration suddenly increased to almost 10% (the 2nd period); this value was maintained for the following 1 h (Figure 6, the 3rd period). The results indicate that ejaculated semen contains a sperm population in which the constituent cells exhibit a chromatin structure or plasma membrane that is vulnerable to osmotic change. A rapid influx of highly concentrated sodium ions through the damaged plasma membrane due to the decline of osmolarity^12^, ^81^ may be a factor that causes DNA breaks during the second period shown in Figure 6.^87^, ^88^ One responsibility of plasma membrane damage with regard to structural chromosome aberrations has been proven in mouse sperm heads that were separated from the tails and injected into oocytes by ICSI.^89^ In normozoospermic males who had experienced repeated ICSI failures, semen dilution and centrifugation with sperm preparation medium increased the positive rates in the TUNEL assay.^33^ This potential membrane damage may be responsible for the individual differences (maximum 20%) in the incidence of structural chromosome aberrations, as demonstrated by a sperm chromosome assay in IVF cases.^3^

#### 4.2.3 ROS

During the next period (Figure 6, the 4th period), other environmental or physiological factors can cause damage to sperm DNA. The incidence of structural chromosome aberrations increased by 5% in 30 min (from 2 to 2.5 h after the removal of the seminal plasma), as determined by the human sperm chromosome assay with ICSI. It has also been reported in mouse and human sperm that incubation times significantly correlate with DNA breaks.^88^, ^90^, ^91^ These facts show that such physiological changes occurring during sperm incubation can gradually induce DNA breaks in mammalian sperm. ROS accompanying ATP production in mitochondria are the responsible factors during this period when SOD derived from the seminal plasma had already been dispersed.^92^, ^93^ Hughes et al.^94^ demonstrated the production of ROS during sperm preparation and showed that the separate in vitro supplementation of antioxidant ascorbate, urate, and alpha tocopherol had beneficial effects on the integrity of sperm DNA.

### 4.3 Fertilization and DNA replication

In general, fertilization is determined by fusion of the plasma membranes of the sperm and oocyte^95^ (Figure 5C and D). The sperm nucleus is freed from the plasma membrane on the oocyte surface and invades into the oocyte cytoplasm; this is followed by decondensation when the nuclear membrane disappears. DNA synthesis has been observed in both male and female pronuclei in murine oocytes at a point 2 h after fertilization.^96^

#### 4.3.1 DNA repair capacity

In the DNA synthesis (S) phase, DNA strand breaks that accumulate in the sperm nucleus are modified by oocyte DNA repair enzymes. Some of these breaks are repaired correctly or mis‐repaired (Figure 5D). The activity of the oocyte DNA repair enzymes was demonstrated in mammalian embryos cultured in the presence of DNA repair inhibitors. These inhibitors were shown to significantly increase the incidence of structural chromosome aberrations in human and murine sperm that had penetrated murine oocytes.^70^, ^97^ Furthermore, in nonhomologous end joining‐defective female mice, there was a significant increase of the metaphases with chromosome aberrations in 1‐cell embryos which had been fertilized with sperm exposed to radiation.^98^ However, in general, DNA repair activity appears to be considerably lower in the mammalian oocyte as compared with that in somatic cells. DNA breaks induced by the same does of mutagenic chemicals or radiation are more frequently converted to structural chromosome aberrations in sperm DNA than somatic culture cell DNA.^38^, ^46^, ^54^ Moreover, most of the chemically or radiation‐induced aberrations in sperm are chromosome‐ or chromatid‐type breaks, thus suggesting that DNA replication has occurred before the sites of broken DNA strands is repaired. In somatic cells, chromatid‐type exchange is generally predominant structural chromosome aberration since the higher activity of DNA repair enzymes modifies most of the sites of DNA breaks.^99^ The lower DNA repair activity in oocytes appears to play a role in which the embryos penetrated by sperm with DNA damage were positively eliminated before and after implantation.

#### 4.3.2 In vitro embryo culture

**FIGURE 7:**
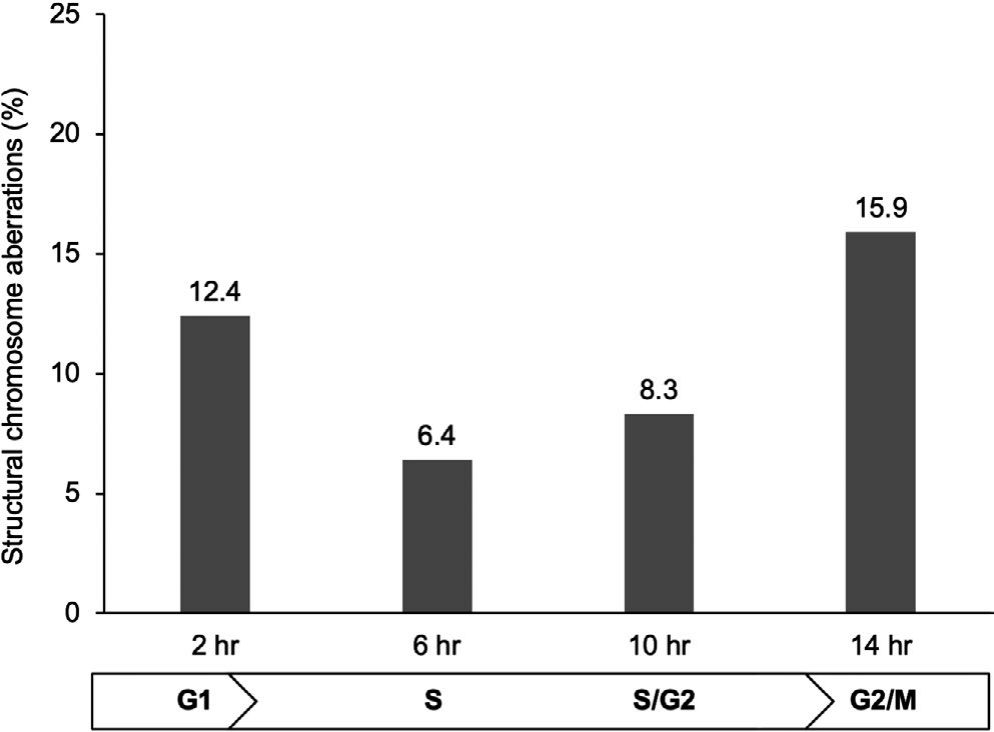
Increases in structural chromosome aberrations in hamster male pronuclei in vitro. Syrian hamster 1‐cell embryos fertilized in vivo were collected and cultured in vitro. Chromosomal analysis of the male pronuclei was conducted at the first cleavage metaphase. The incidence of structural aberrations in the chromosome complements of the male pronuclei were significantly higher in the hamster oocytes transferred to in vitro culture conditions at the G1/S and G2/M cell cycle stages (2 and 14 h after fertilization, respectively)

In the Syrian hamster, chromosomal fragmentations can be induced in the sperm chromosomes of 1‐cell embryos under in vitro culture conditions (my own unpublished data, Figure 7). When Syrian hamster embryos that were fertilized in vivo were collected from the oviducts and then cultured in vitro to the first cleavage metaphase, a significant increase of structural chromosome aberrations was found in the sperm chromosome complements of the embryos that were transferred to in vitro culture at the time of sperm decondensation (0–2 h after fertilization (G1/S phase), 12.4%) or male pronucleus condensation (18–20 h after fertilization (G2/M phase), 15.9%). These are phases when the structure of the sperm chromatin alters drastically.^96^ Although the mechanism of this phenomenon has yet to be investigated, the DNA repair activity of Syrian hamster oocytes may decrease under in vitro culture conditions. It is well known that the early cleavage stage embryos of this species are very vulnerable to in vitro culture conditions.^100^, ^101^ Since the DNA repair system of human oocytes has yet to be investigated in detail, there may be cell cycle‐dependent phases when human oocytes become sensitive to in vitro culture conditions. Time lapse observation appears to be effective in identifying the reduction of developmental potency during in vitro culture conditions.

## 5 DNA DAMAGE IN EJACULATED SPERM VERSUS TESTICULAR SPERM

Azoospermia is the term used for infertile males in which no spermatozoa is found in the sediment of centrifuged semen samples.^71^, ^102^ The approximate frequency of azoospermic males estimated in the general population of England was reported to be 2%.^103^ With regard to the ART treatment of infertile males with azoospermia, there have been concerns about the DNA integrity of ejaculated spermatozoa. Higher pregnancy and delivery rates in ICSI have been reported with testicular sperm by several investigators as compared with ejaculated sperm.^104^, ^105^, ^106^, ^107^, ^108^, ^109^, ^110^, ^111^, ^112^ In addition, Mehta et al.^113^ and Moskovtsev et al.^114^ found comparably lower DNA damage in testicular sperm and concluded that testicular sperm extraction (TESE) can be considered for oligozoospermic men who have elevated levels of TUNEL‐positive ejaculated sperm. These findings provide a reason for the selection of TESE for the treatment of azoospermic males, even if some sperm are found in their ejaculated semen. When reviewing previous literature, I found that there are several factors that could affect distinct pregnancies or outcomes when compared between ICSI with testicular sperm and ejaculated sperm. For example, in the studies reported by Bendikson et al.^115^ and Amirjannati et al.,^116^ centrifugation at very high speeds (1500–3000 *g* and 1000 *g*, respectively) were applied to ejaculated sperm. Hauser et al.^117^ showed that the freezing and thawing process, which is generally recommended to prevent ICSI procedures from being canceled,^118^ were harmful to both testicular and ejaculated sperm. As described earlier, the cryopreservation of human sperm can result in damage to the plasma membrane and can significantly induce DNA breaks; this is because the nucleus is exposed to significant intracytoplasmic osmotic changes.^12^, ^33^ Accordingly, it is difficult to maintain the DNA integrity of ejaculated sperm from azoospermic males during sperm preparation, which may require dilution and centrifugations with different liquids. Considering these factors, Ohno et al.^119^ carefully prepared fresh or frozen ejaculated sperm from males with severe cryptozoospermia with a micromanipulator and froze the sperm cells with a sucrose base cryoprotectant‐free medium and consequently obtained better outcomes with ICSI.

## 6 CONCLUSION

Previous studies using the sperm chromosome assay have generated very important information that has enabled us to understand the mechanisms by which sperm DNA damage is caused in humans. Consequently, it was revealed that the proportion of sperm showing DNA damage is initially low and there are larger numbers of sperm with potential membrane and DNA damage after ejaculation and separation from the seminal plasma. However, sufficient consideration and discussion relating to such data have not been conducted in previous review articles relating to DNA damage, as determined by direct DNA damage assays. Direct assays for sperm DNA damage use a portion of a whole sperm population, which contains immotile sperm and amorphous head sperm along with motile normal sperm. Therefore, the results obtained are always related to the frequencies of such abnormal sperm, which may not participate in spontaneous fertilization and IVF. For a rise of a successful outcome in the ART treatment, it is important to estimate the genetic integrity of the normal sperm population, although the direct sperm assays are not suitable for the purpose. Especially, we need phenotypes that can be used to estimate the genetic integrity of a sperm which will be selected and injected into an oocyte in the ICSI treatment. Data arising from the sperm chromosome assay appears to provide hints on how to reduce the risk of selecting and using sperm with DNA damage in ICSI. Namely, a sperm that could maintain the integrity of the DNA and plasma membrane should be quickly selected and kept away from the stress caused by environmental conditions and preparatory operations prior to ICSI.

## CONFLICT OF INTEREST

The author declares no conflict of interest.

## HUMAN RIGHTS STATEMENTS AND INFORMED CONSENT

My previous studies that provide unpublished data here were approved by the Institutional Review Boards of Asahikawa Medical College and Hirosaki University Graduate School of Medicine.

## ANIMAL STUDIES

My own unpublished data were from studies conducted in accordance with the ethical standards of the committees in charge of the research institutions.
